# TENT5C functions as a corepressor in the ligand‐bound glucocorticoid receptor and estrogen receptor α complexes

**DOI:** 10.1111/febs.70137

**Published:** 2025-05-27

**Authors:** Yin Li, Lalith Perera, Rebecca S. He, Marine Baptissart, Robert M. Petrovich, Marcos Morgan

**Affiliations:** ^1^ Reproductive and Developmental Biology Laboratory National Institute of Environmental Health Sciences Durham NC USA; ^2^ Genomic Integrity and Structural Biology Laboratory National Institute of Environmental Health Sciences Durham NC USA

**Keywords:** estrogen receptor α, glucocorticoid receptor, LXXLL motif, molecular dynamics, terminal nucleotidyltransferase 5C

## Abstract

Terminal nucleotidyltransferase 5C (TENT5C) is a noncanonical poly(A) polymerase that promotes cancer suppression. TENT5C has been proposed to mediate the susceptibility of multiple myeloma to treatment with dexamethasone, a steroid hormone analog that binds to the glucocorticoid receptor (GR). However, the relationship between TENT5C and nuclear receptor (NR) signaling remains unclear. In this study, we investigate the regulatory role of TENT5C in the GR and estrogen receptor α (ERα) ligand complexes. We find that TENT5C acts as a corepressor of both GR and ERα. Molecular dynamics simulations indicate that the third TENT5C LXXLL motif directly interacts with ERα, but not GR. The physical interaction of TENT5C and ERα is supported by co‐immunoprecipitation assays. Reporter assays show that mutations to the third TENT5C LXXLL motif disrupt TENT5C‐mediated repression of ERα but do not affect the repression of the GR complex. In addition, the disruption of TENT5C poly(A) polymerase activity does not appear to affect TENT5C repression of ERα in the cell lines studied. Taken together, our findings highlight a role of TENT5C as an NR corepressor, differentially modulating GR‐ and ERα‐induced transcriptional activity.

## Graphical abstract

TENT5C is a suppressor of ERα and GR transcriptional activation. A specific LXXLL motif in TENT5C, absent in the other members of the TENT5 subfamily, mediates a stable interaction with the Ligand Binding Domain of ERα but not with that of GR. Mutations to the LXXLL motif result in a weaker interaction between TENT5C and ERα and rescue ERα transcriptional activation in a context‐dependent manner.

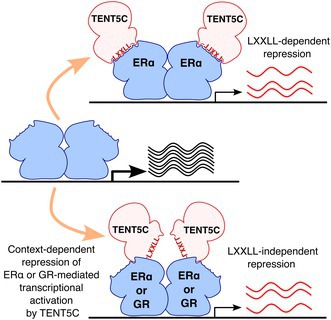

**Abbreviations**
C3: complement C3
DEX: dexamethasone
E2: 17β‐estradiol
ERE: estrogen response element
ERα: estrogen receptor α
FKBP5: FKBP prolyl isomerase 5
GR: glucocorticoid receptor
GRE: glucocorticoid response element
LBD: ligand binding domain
MD: molecular dynamics
MM: multiple myeloma
MMGBSA: molecular mechanics with generalized Born and surface area solvation
NR: nuclear receptor
PGR: progesterone receptor
RMSD: root mean squared deviation
RMSF: root mean squared fluctuations
TENT5C: terminal nucleotidyltransferase 5C
TRBP: transactivation response RNA binding protein

## Introduction

Terminal nucleotidyltransferase 5C (TENT5C) is a noncanonical poly(A) polymerase required for fertility and different aspects of hematopoiesis [1, 2, 3, 4, 5, 6]. TENT5C deleterious mutations are highly prevalent in multiple myeloma (MM) [7, 8]. A common treatment for MM is the administration of dexamethasone (DEX), an analog of the steroid hormone glucocorticoid. Exposure to DEX triggers apoptosis of the cancerous cells, a process proposed to be modulated by TENT5C [9, 10]. Also, the absence of TENT5C has been shown to alter the estrogen receptor (ER) pathway [9].

Steroid hormones regulate fertility, metabolism, and innate immunity, among other physiological functions. These activities are mediated by specific nuclear receptors (NR). The glucocorticoid receptor (GR) is primarily implicated in the immune response but is also critical for several other processes such as metabolic regulation and cognitive behavior [11]. As such, administration of DEX is a standard treatment to reduce inflammation. Estrogen receptor α (ERα) and estrogen receptor β (ERβ) are required for normal fertility, but they also differentially regulate lymphopoiesis [12, 13, 14, 15]. ERα and ERβ have partially overlapping sets of target genes which can in part explain their specific functions [16, 17, 18].

Binding of the nuclear receptors (NRs) to their steroid ligands induces their translocation to the nucleus where they can directly bind to specific response elements to modulate the transcription of target genes. The activity of the NRs is affected by their interaction with coregulators, which could either activate (coactivators) or repress (corepressors) the expression of target genes [19]. Coactivators and corepressors can be common to multiple receptors, which leads to crosstalk between different steroid hormone pathways [19, 20, 21, 22]. For example, ER and progesterone receptor activation was initially shown to be regulated by the availability of steroid receptor coactivator‐1 for which the NRs compete [20]. Later, the nuclear receptor corepressor complex was also shown to be a limiting factor regulating NR activity [22].

NRs have a DNA binding domain that allows each receptor to interact with specific DNA sequences. For GR and ERs, these target sequences are referred to as glucocorticoid response element (GRE) and estrogen response element (ERE), respectively. The ligand binding domain (LBD) of the NRs, in addition to binding to specific steroid hormones, can also interact with coregulators. In particular, the LBD mediates the interaction with coregulators in a ligand‐dependent manner. The LXXLL amino acid motif, commonly found in coregulators, is one of the motifs frequently bound by the LBD [23, 24]. In contrast, an N‐terminal activating region interacts with coregulators in a ligand‐independent manner [25].

In this study, we investigate the role of TENT5C in NR activity modulation. Using reporter assays, we showed that TENT5C reduces the GR‐ and ERα‐mediated transcriptional activities. Mutagenesis analysis and molecular dynamics (MD) simulations showed that TENT5C interacts with ERα LBD through the LXXLL coregulator motif. However, this motif is not required for TENT5C's interaction with GR and the inhibition of GR transcriptional activity. At the same time, the poly(A) polymerase activity of TENT5C is not required for repressing ERα‐mediated transcriptional activation. In summary, our work demonstrates that TENT5C is an NR corepressor regulating GR or ERα complexes through different mechanisms.

## Results

### TENT5C is a corepressor of GR and ERα

**Fig. 1:**
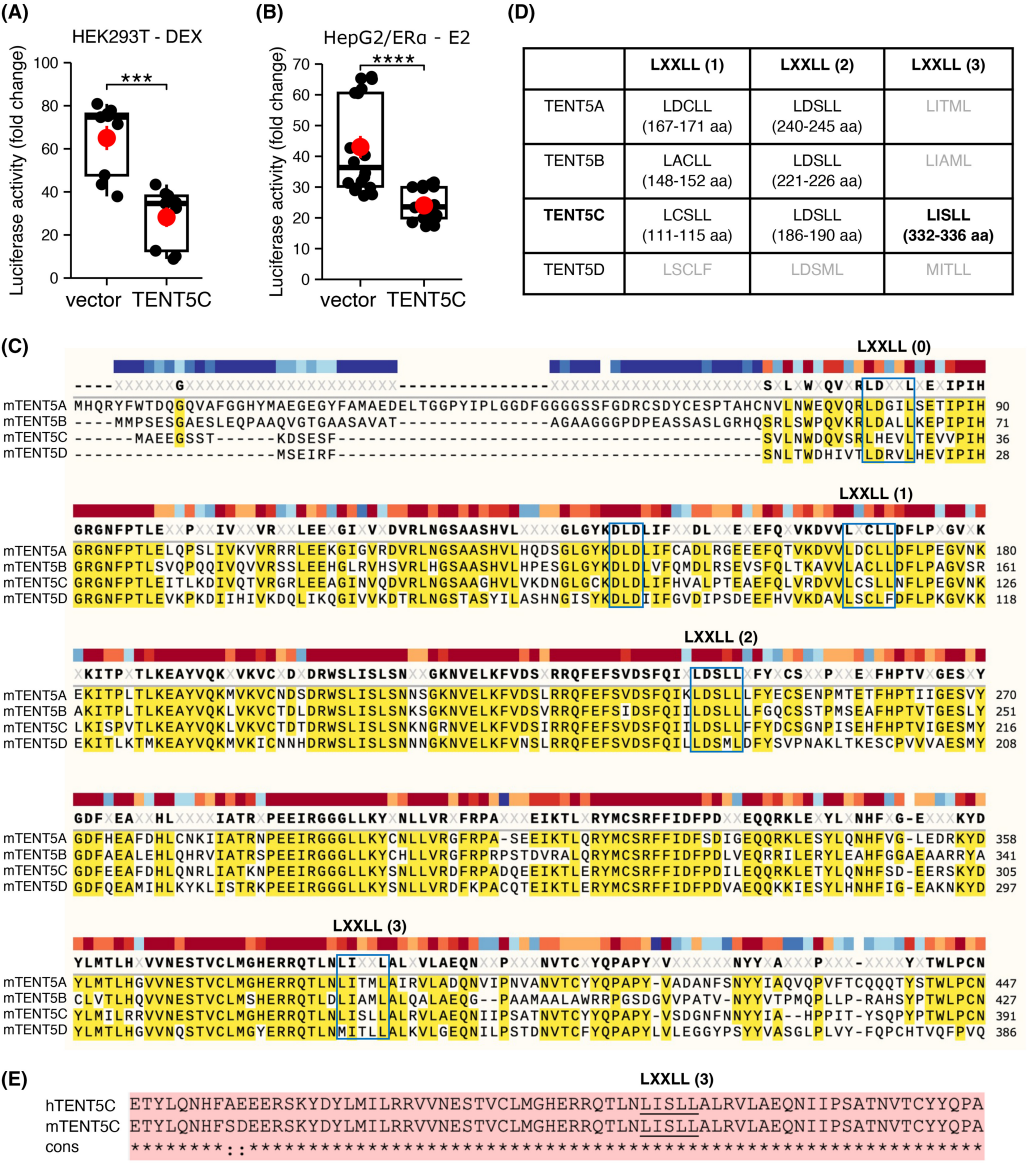
TENT5C reduces GR‐ and ERα‐mediated transcriptional activation and has a characteristic C‐terminal LXXLL motif. (A) Box plot of GRE‐luciferase reporter assays in HEK293T cells. Cells were transiently transfected with 2xGRE‐luc, pRL‐TK‐luc, GR, and TRBP with vector or TENT5C. After 30 h, cells were treated with vehicle or 100 nm DEX for 18 h (*n* = 9). (B) Box plot of ERE‐luciferase reporter assay in HepG2/ERα cells. Cells were transiently transfected with 3xERE‐luc and pRL‐TK‐luc, together with vector or TENT5C. After 30 h, cells were treated with vehicle or 10 nm E2 for 18 h (*n* = 18). Fold increases were calculated relative to each vehicle treatment and compared using the Dunn test. Data points indicate the values of biological replicates. The box plot center bar represents the median. The upper and lower sides of the box plot represent the first and third quantiles, respectively. The lower and upper whiskers represent the tenth and ninetieth percentiles. The mean is indicated by a red dot and the standard error by a red bar. ***, *P* < 0.001 and ****, *P* < 0.0001. (C) Multiple protein alignment using Muscle of mouse TENT5A (NP_001153850), TENT5B (NP_780516), TENT5C (NP_001136424), and TENT5D (NP_001156576.1) highlighting the position of the LXXLL motifs. The aspartic acids critical for the poly(A) polymerase activity of TENT5C are also indicated. The sequences were downloaded from the National Center for Biotechnology Information (NCBI). (D) Summary table of the LXXLL motifs present in the different TENT5 proteins. (E) Sequence alignment using Muscle of human (NP_060179) and mouse (NP_001136424) TENT5C proteins showing high sequence identity and homology in the LXXLL (3) motif region. The sequences were downloaded from NCBI.

Although TENT5C has been implicated in the response to DEX in MM, the impact of TENT5C on GR functions remains poorly understood [9, 10]. To investigate the regulatory activity of TENT5C in response to DEX, we performed a GR reporter assay using a luciferase gene under the control of a GRE‐containing promoter. When cotransfected with GR and transactivation response RNA binding protein (TRBP), a known coactivator of GR [26] in HEK293T cells, we found that TENT5C significantly reduced GRE‐mediated GR transcriptional activity in the presence of DEX (Fig. 1A). This result indicates that TENT5C is a GR corepressor.

TENT5C has also been implicated in regulating the estrogen response in MM [9]; therefore, we speculated that it could also be a coregulator of ERs. Given the already established mechanistic insights on the role of ERα regulatory domains in immune cell development, we decided to focus on this particular estrogen receptor [14]. To evaluate the impact of TENT5C on the ERα transcriptional activity, we used a luciferase reporter containing the ERE. When TENT5C was transfected in HepG2/ERα cells, a cell line stably expressing ERα [27], there was a significant reduction in the ERE‐mediated ERα transcriptional activity by TENT5C in the presence of 17β‐estradiol (E2) (Fig. 1B). Taken together, the data suggest that TENT5C is a corepressor of both GR and ERα signaling.

### A TENT5C‐specific LXXLL motif is predicted to bind stably to ERα but not GR

To further investigate the role of the TENT5C as an NR coregulator, we searched for the LXXLL coregulator motif in the mouse TENT5 subfamily [1, 28]. TENT5A, TENT5B, and TENT5C have two shared LXXLL motifs: LXXLL (1) and LXXLL (2). TENT5B has a specific LXXLL motif close to its N‐terminal end (LXXLL (0)), while TENT5C has an additional one close to its C‐terminal end (LXXLL (3)) (Fig. 1C). No LXXLL motifs are present in TENT5D (Fig. 1C). The location of the LXXLL sequences is summarized in Fig. 1D. Since the X‐ray crystal structure of human TENT5C containing all three LXXLL motifs is available (pdb ID: 6w36; Fig. S1) [29], we used MD simulations to further investigate the potential interactions of TENT5C LXXLL motifs with ERα and GR. Note that the human and mouse TENT5C protein sequences share a high sequence identity (Fig. 1E).

**Fig. 2:**
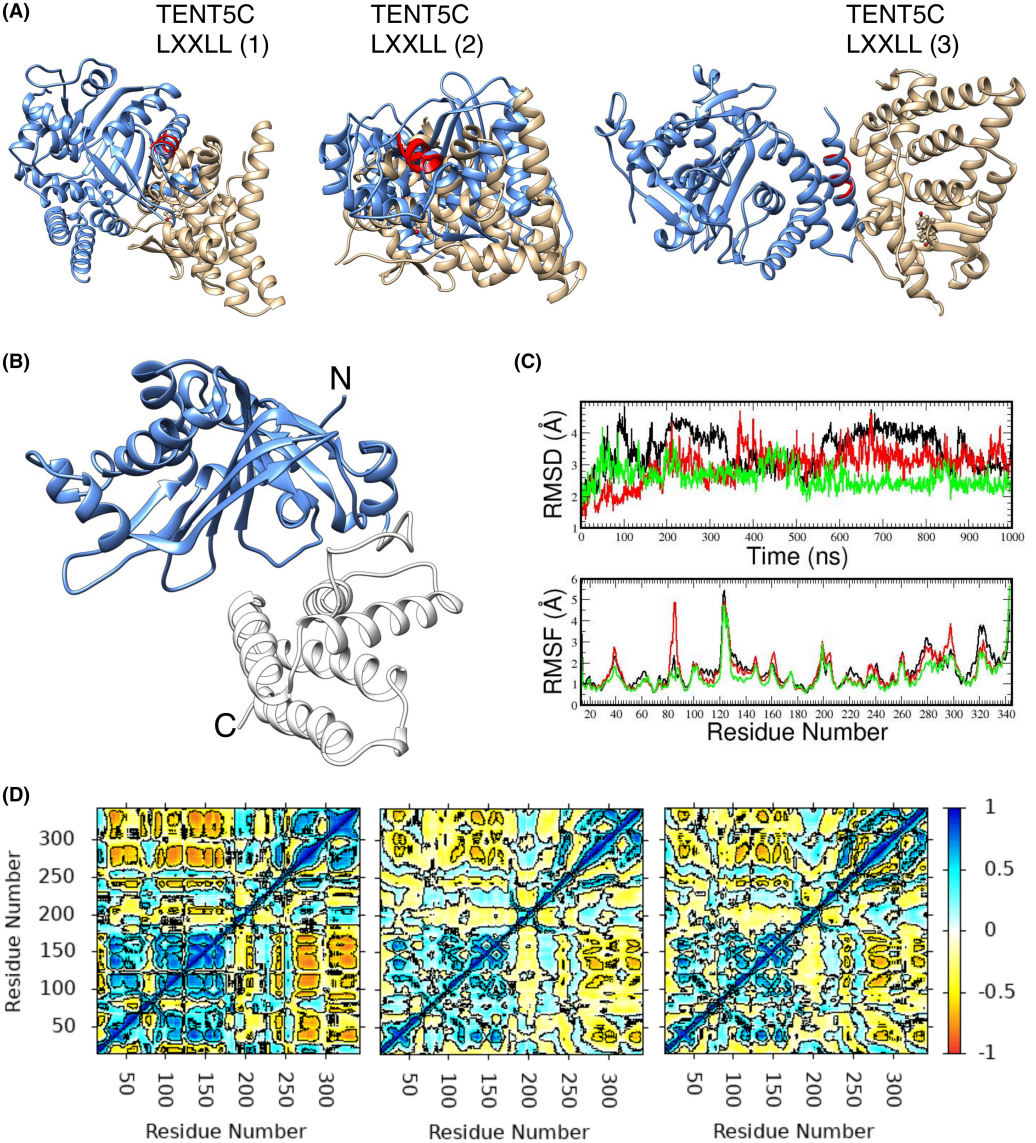
TENT5C LXXLL (3) motif is predicted to interact with ERα. (A) Possible conformations of the TENT5C/ERα complex generated with chimera‐1.16 based on the alignment of three LXXLL motifs of TENT5C with that of the ERα‐bound peptide found in the crystal structure. Panels correspond to the use of the LXXLL (1) motif (*left*), LXXLL (2) motif (*middle*), and LXXLL (3) motif (*right*) in ERα interactions. ERα is shown in wheat and TENT5C in blue. E2 is also shown bound to ERα as well as the LXXLL‐containing peptide in red. (B) Ribbon diagram of TENT5C with its two regions highlighted in white and blue generated with chimera‐1.16. (C) Root mean squared deviations (RMSD) calculated from three independent simulations of TENT5C are shown in the top panel. The root mean squared fluctuations (RMSF) of backbone atoms averaged over the entire microsecond for each of these three different trajectories of TENT5C are shown in the lower panel. Colors black, red, and green correspond to each trajectory. (D) Dynamic cross‐correlations of alpha carbons calculated from each microsecond trajectory of TENT5C averaged over the entire microsecond. Each panel corresponds to an independent trajectory.

We next used an X‐ray structure of ERα bound to a LXXLL‐containing peptide to align each of the three LXXLL motifs of TENT5C on ERα by superimposing each motif to the bound peptide [30]. The binding of both the LXXLL (1) and LXXLL (2) motifs to ERα run into catastrophic situations in which segments of TENT5C significantly overlap with the area already occupied by ERα (Fig. 2A). Only the LXXLL (3) motif shows no steric clashes while bound to ERα, suggesting a potential interaction mediated by the LXXLL (3) motif of TENT5C and the LBD of ERα (Fig. 2A). Because the TENT5C LXXLL (1) and (2) motifs are partially located in helices, we examined whether they could be dynamically folded into exposed configurations suitable for NR binding. To explore these possibilities, we carried out MD simulations of solvated TENT5C. The root mean squared deviation (RMSD) analysis showed that TENT5C displays a rather dynamic but stable conformation in solution when compared to the initial crystal structure (Fig. 2B; Fig. S1) [29]. Root mean squared fluctuations (RMSF) of the residue backbones depict stable LXXLL (1) and (2) motifs, as shown by the small RMSF values in the regions 113–118 and 188–192 (Fig. 2C). Instead, the LXXLL (3) motif, located in the region 332–336, already in a favorable ERα bound conformation, showed more dynamics conducive to establishing interactions with NRs (Fig. 2C). Dynamics cross‐correlations further confirm that the LXXLL (1) and (2) motifs follow consorted motion with their interacting residues within the TENT5C protein, incompatible with exposed conformations amenable to NR interactions (Fig. 2D). Therefore, as predicted by the initial alignments with the X‐ray structure, the LXXLL (1) and LXXLL (2) motifs do not adopt conformations that can show binding to the LBD of NRs.

**Fig. 3:**
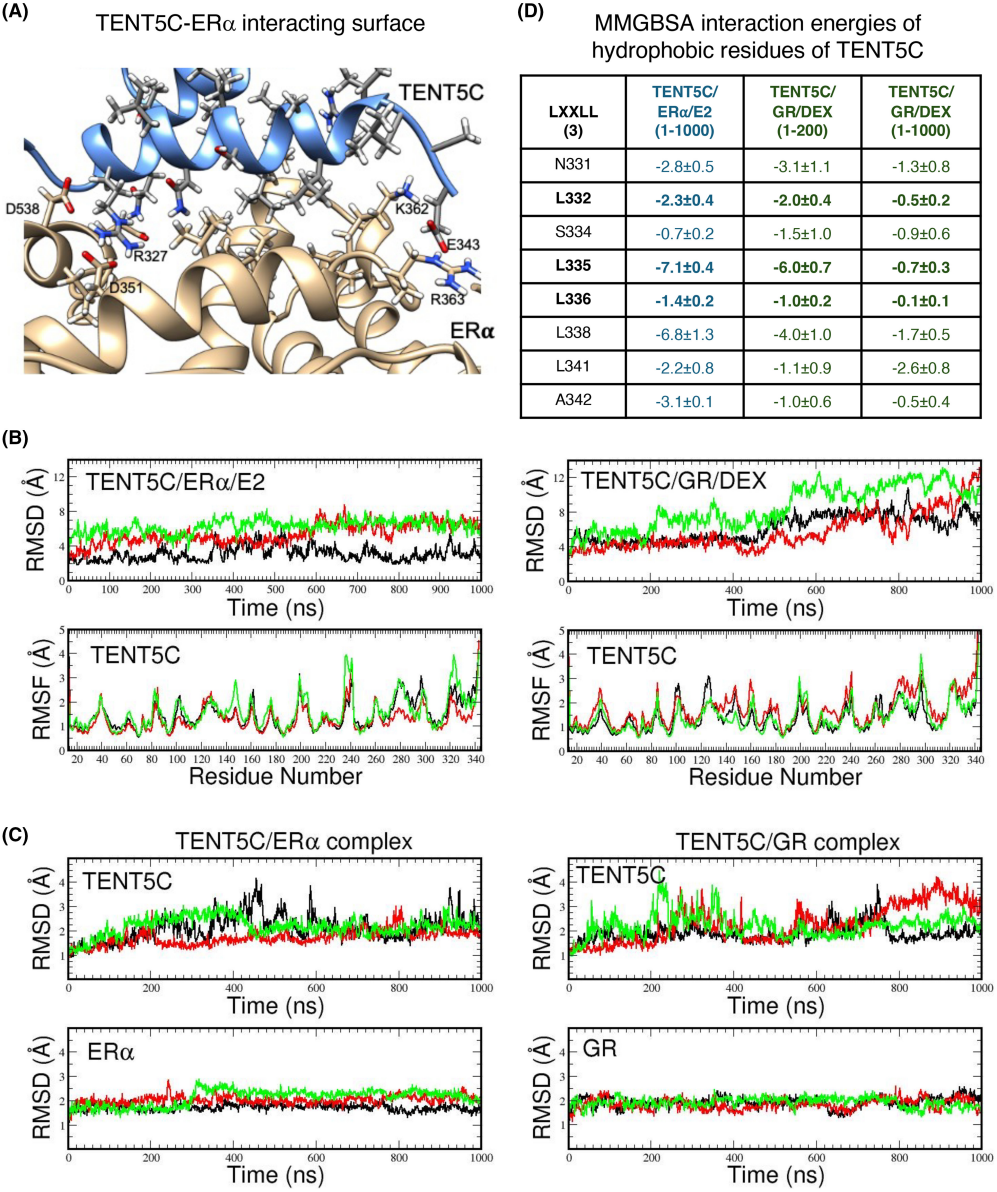
TENT5C is predicted to form stable interactions with E2 bound ERα. (A) The interacting surface of TENT5C and ERα from a representative solution structure of the TENT5C/ERα/E2 complex generated with chimera‐1.16. TENT5C is shown in blue and ERα in wheat. (B) Root mean squared deviations (RMSD, *top*) and root mean squared fluctuations (RMSF, *bottom*) of TENT5C/ERα/E2 (*left*) and TENT5C/GR/DEX (*right*) complexes. RMSD values were calculated by aligning each configuration at each nanosecond time interval with the starting conformation. RMSF results are presented only for TENT5C. The three independent molecular dynamics (MD) trajectories are shown in black, red, and green. (C) RMSD of TENT5C (*top*) and the respective nuclear receptors (*bottom*) from the TENT5C/ERα/E2 (*left*) or the TEN5C/GR/DEX (*right*) complexes (*n* = 3). (D) Table of molecular mechanics with generalized Born and surface area solvation (MMGBSA) energy analysis for TENT5C critical hydrophobic residues in the TENT5C/ERα/E2 and TENT5C/GR/DEX complexes. Leucine residues within the LXXLL motif are in bold. Since TENT5C/GR/DEX complexes dissociate, the early (200 ns) of the simulations were used to estimate energies when TENT5C and GR were in reasonable contact.

**Fig. 4:**
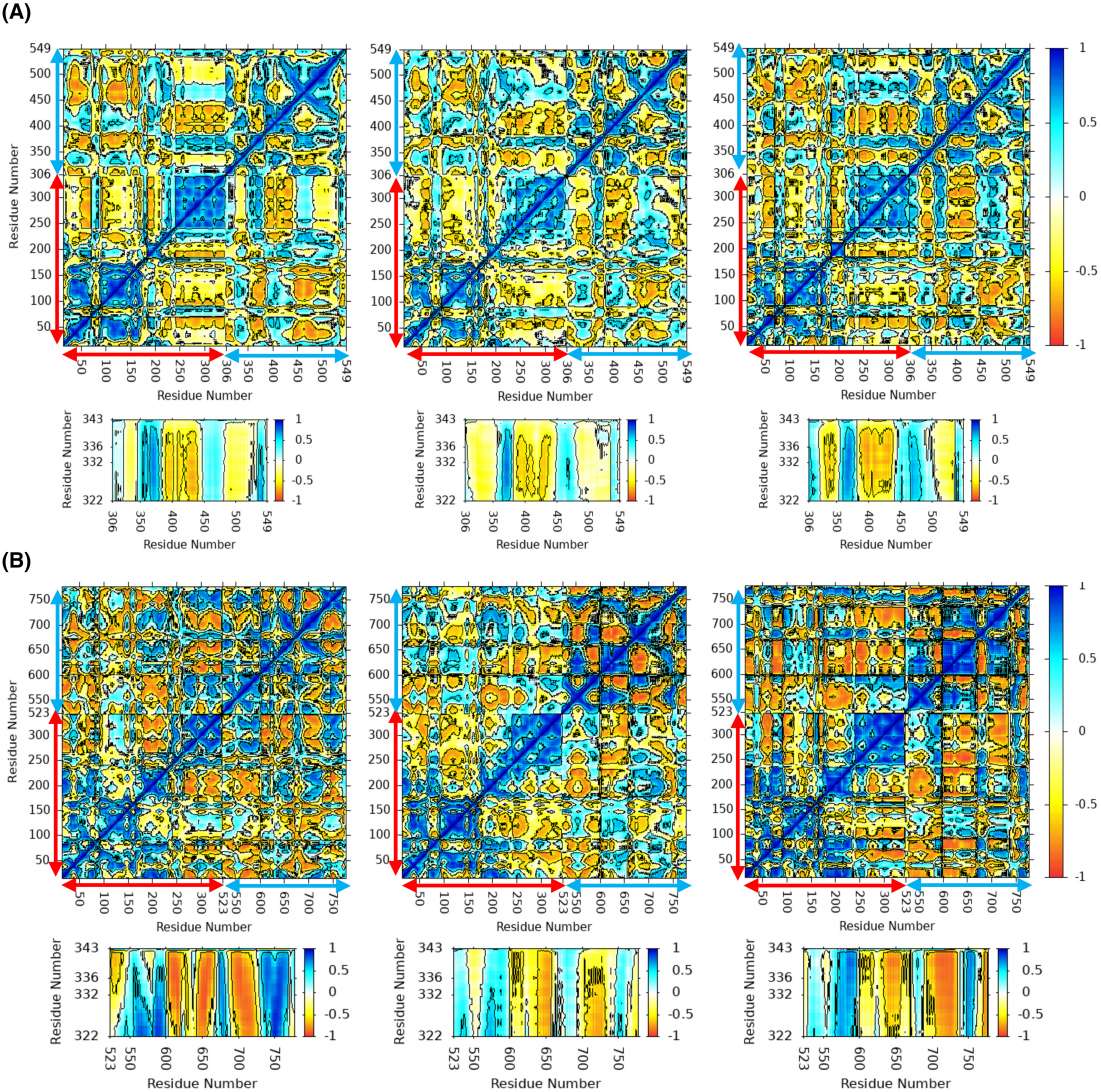
Correlated motions of TENT5C/ERα/E2 or TENT5C/GR/DEX complexes. (A) Dynamic cross‐correlations calculated from alpha carbons of the TENT5C/ERα/E2 complex. Panels correspond to the three independent simulations of TENT5C/ERα/E2 complexes. The red arrows mark the TENT5C residues, while the blue ones mark the ERα residues. The bottom panels show the correlated motion of LXXLL (3) (residues 332–336) with the ERα residues in the helices 3 (residues 355–362), 5 (residues 372–380), and 12 (residues 538–545). (B) Dynamic cross‐correlations calculated from alpha carbons of the TENT5C/GR/DEX complex. Panels correspond to the three independent simulations of TENT5C/GR/DEX complexes. The red arrows mark the TENT5C residues, while the blue ones mark the GR residues. The bottom panels show the correlated motion of LXXLL (3) (residues 332–336) with the GR residues in helices 3 (residues 572–579), 5 (residues 589–597), and 12 (residues 752–759).

The structures of ERα and GR binding to TENT5C via the LXXLL (3) motif were further scrutinized through microsecond MD simulations (Fig. 3A; Fig. S2). Stable bound complexes were observed through root mean squared deviations (RMSD) and RMSF for TENT5C interaction with ERα, but not GR (Fig. 3B,C). Molecular mechanics with generalized Born and surface area solvation (MMGBSA) interaction‐free energies calculated at the residue level using the conformations from the MD simulations for residues in the LXXLL (3) motif along with some important hydrophobic residues around this motif are given in Fig. 3D. TENT5C hydrophobic residues L332 and L335 in the LXXLL motif are actively participating in the interactions with the ERα coregulator binding residues (Fig. 3D). In addition to the LXXLL motif accessibility, several flanking residues are critical to establish and maintain an interaction with NRs. Among them, R327 (−29.0 ± 2.7 kcal·mol^−1^) on the N‐terminal side makes salt bridges with D538 (−23.0 ± 2.1 kcal·mol^−1^) as well as with D351 (−13.0 ± 1.9 kcal·mol^−1^) of ERα, further strengthening the protein interaction. Similarly, E343 (−35.4 ± 44.9 kcal·mol^−1^) of TENT5C that is in a mobile loop area can be in contact with K362 (−21.2 ± 2.7 kcal·mol^−1^) and R363 (−12.6 ± 2.6 kcal·mol^−1^) residues of ERα additionally strengthening the C‐terminal interactions (Fig. 3A). Instead, for GR, the interaction with the LXXLL (3) motif and its flanking regions diminishes as the simulation progresses (Fig. 3D). The dynamic cross‐correlations confirm that the LXXLL (3) motif is in a concerted motion with helix 3, 5, and 12 of ERα (Fig. 4A; Videos [Link], [Link]). A less concerted motion was observed with GR (Fig. 4B). Taken together, these results indicate that GR and ERα can directly interact with TENT5C LXXLL (3) motif, but the LXXLL (3) flanking regions only support a stable binding to ERα.

### TENT5C LXXLL (3) motif is critical for the interaction of TENT5C and ERα

**Fig. 5:**
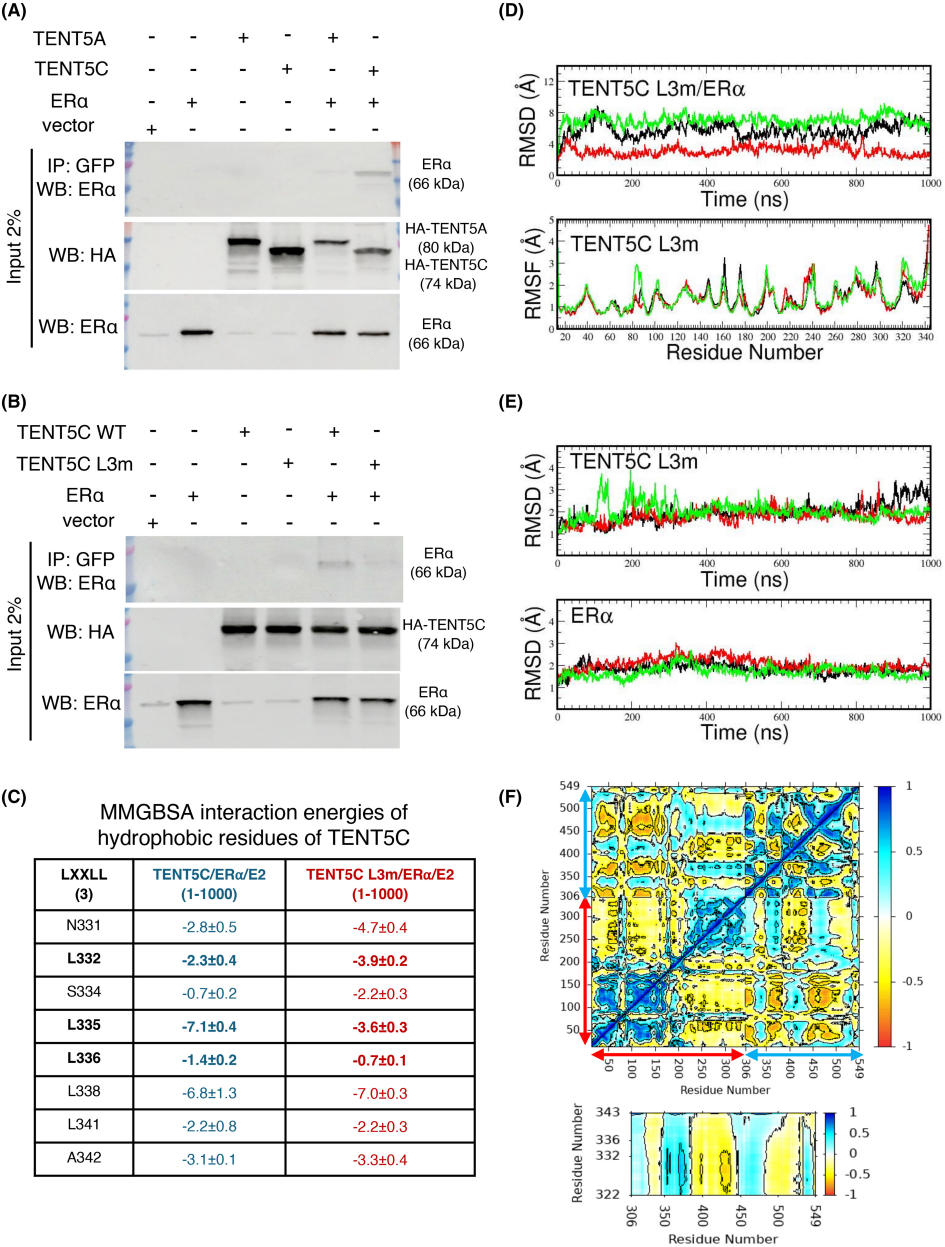
TENT5C LXXLL (3) motif participates in the formation of the TENT5C/ERα complex. (A) Co‐immunoprecipitation (Co‐IP) assay of TENT5C with ERα. HEK293T cells were cotransfected with different combinations of vector, ERα, TENT5A, or TENT5C for 30 h. Cell lysates were prepared in the IP lysis buffer and immunoprecipitated using GFP nanobody for 2 h. The presence of HA‐tagged TENT5A, TENT5C, or ERα in 2% of input samples was detected by anti‐HA tag or anti‐ERα antibody. The presence of ERα in the precipitants was detected by anti‐ERα antibody (*n* = 3). (B) Co‐IP assay of TENT5C WT or TENT5C L3m (LXXAA) with ERα. HEK293T cells were cotransfected with different combinations of vector, ERα, TENT5C WT, or TENT5C L3m for 30 h. Cell lysates were prepared in the IP lysis buffer and immunoprecipitated using GFP nanobody for 2 h. The presence of HA‐tagged TENT5C WT, TENT5C L3m, or ERα in 2% of input samples was detected by anti‐HA Tag or anti‐ERα antibody. The presence of ERα in the precipitants was detected by anti‐ERα antibody (*n* = 2). (C) Table of molecular mechanics with generalized Born and surface area solvation (MMGBSA) interaction energies for TENT5C WT (as shown in Fig. 3D) and TENT5C L3m critical hydrophobic residues. (D) RMSDs of TENT5C L3M/ERα/E2 complex (*top*) and RMSF of TENT5C L3m within the complex (*bottom*) (*n* = 3). (E) RMSD values of TENT5C L3m (*top*) and ERα (*bottom*) within the TENT5C L3M/ERα/E2 complex (*n* = 3). (F) Representative dynamic cross‐correlations calculated from alpha carbons of the TENT5C L3m/ERα/E2 complex. The red arrows mark the TENT5C L3m residues, while the blue ones mark the ERα residues. The bottom panels show the correlated motion of LXXAA (3) (residues 332–336) with the ERα residues in helices 3 (residues 355–362), 5 (residues 372–380), and 12 (residues 538–545).

MD simulations support a direct binding between TENT5C and ERα through the LXXLL (3) motif. To test this interaction directly, we next used a double GFP‐ and HA‐tagged TENT5C protein for pull‐downs in the absence of reliable antibodies. We also tagged TENT5A as a control, which lacks the LXXLL (3) motif. While we found ERα present in the TENT5C pull‐down, TENT5A failed to precipitate ERα robustly (Fig. 5A). Thus, ERα strong interaction with TENT5C, but not TENT5A, supports a binding mediated by the LXXLL (3) motif. To further evaluate whether the interaction between TENT5C and ERα is mediated by the LXXLL (3) motif, we mutated the last two leucines of the motif to alanines (LXXAA, L3m). Immunoprecipitation assays showed an interaction of ERα with TENT5C WT but not with TENT5C L3m further supporting a direct interaction between TENT5C and ERα through the LXXLL (3) motif (Fig. 5B). Unexpectedly, we found that TENT5C L3m was predicted to interact with similar strength with ERα according to MMGBSA interaction energies, RMSD, RMSF, and dynamic cross‐correlations (Fig. 5C–F). We speculate that the discrepancy between the IP data and the simulations could be due to a reduction in the formation of the stable complexes.

### TENT5C LXXLL (3) motif is critical for specifically repressing the ERα‐mediated transcriptional activation

**Fig. 6:**
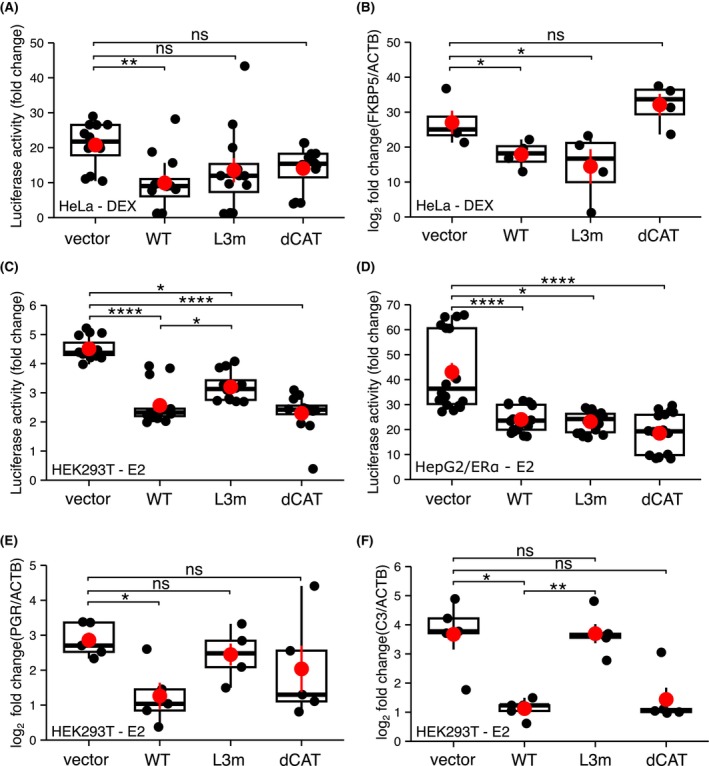
TENT5C LXXLL (3) motif is required for the suppression of ERα‐mediated transcriptional activation in a cell‐type specific manner. (A) Box plot of GRE‐luciferase reporter activity in the presence of TENT5C WT, TENT5C L3m, or TENT5C dCAT expression vectors in HeLa cells (*n* = 12). Cells were transiently transfected with 2xGRE‐luc, pRL‐TK (internal control), and TRBP, together with the expression plasmids as shown in the graphs. After 30 h, cells were treated with vehicle or 100 nm DEX for 18 h. Fold increases were calculated relative to each vehicle treatment and compared using the Dunn test. (B) Box plot of *FKBP5* expression quantified by qPCR. HeLa cells were transiently transfected with vector, TENT5C WT, TENT5C L3m, or TENT5C dCAT expression plasmids (*n* = 4). After 24 h, cells were treated with vehicle or 100 nm DEX for 18 h. The expression level of *FKBP5* was normalized to that of *ACTB*. Log2 fold changes are relative to corresponding vehicle‐treated samples and are compared using a paired *t*‐test. (C) Box plot of ERE‐luciferase reporter activity in the presence of TENT5C WT (*n* = 15), TENT5C L3m (*n* = 12), TENT5C dCAT (*n* = 12), or expression vector (*n* = 15) in HEK293T cells. Cells were transiently transfected with 3xERE‐luc, pRL‐TK (internal control), together with expression plasmids as shown in the graphs. After 30 h, cells were treated with vehicle or 10 nm E2 for 18 h. Fold increases were calculated relative to each vehicle treatment and compared using the Dunn test. *P*‐values were adjusted for multiple comparisons using Hommel's correction. (D) Box plot of ERE‐luciferase reporter activity in the presence of TENT5C WT, TENT5C L3m, or TENT5C dCAT expression vectors in HepG2/ERα cells (*n* = 18). Cells were transiently transfected with 3xERE‐luc, pRL‐TK (internal control), together with expression plasmids as shown in the graphs. After 30 h, cells were treated with vehicle or 10 nm E2 for 18 h. Fold increases were calculated relative to each vehicle treatment and compared using the Dunn test. The vector and TENT5C WT data were shown in Fig. 1B. (E, F) Box plot of *PGR* (E) and *C3* (F) expression quantified by qPCR. HEK293 cells were transiently transfected with ERα and TENT5C WT, TENT5C L3m, or TENT5C dCAT expression plasmids (*n* = 5). After 24 h, cells were treated with vehicle or 10 nm E2 for 18 h. The expression levels were normalized to that of *ACTB*. Log2 fold changes are relative to corresponding vehicle‐treated samples and are compared using a paired *t*‐test. *P*‐values were adjusted for multiple comparisons using Hommel's correction. Data points indicate the values of biological replicates. The box plot center bar represents the median. The upper and lower sides of the box plot represent the first and third quantiles, respectively. The lower and upper whiskers represent the tenth and ninetieth percentiles. The mean is indicated by a red dot and the standard error by a red bar. ns, not significant; *, *P* < 0.05; **, *P* < 0.01; and ****, *P* < 0.0001.

Although the MD simulation‐predicted interaction between TENT5C and GR is unstable, the proteins can still interact through the LXXLL (3) motif. To investigate whether the LXXLL (3) motif could regulate the TENT5C‐mediated corepression of GR, we repeated our reported assays in the presence of TRBP with TENT5C WT and TENT5C L3m mutant proteins. As suggested by the MD simulation results, the TENT5C L3m mutant still repressed the activity of the GR reporter (Fig. 6A); thus, the LXXLL (3) motif appears to be insufficient to promote a stable interaction between TENT5C and GR. To investigate the role of the TENT5C catalytic activity on GR transcriptional inhibition, we evaluated the expression of an endogenous GR target. TENT5C WT and L3m proteins repressed the DEX‐induced activation of FKBP prolyl isomerase 5 (*FKBP5*). However, the TENT5C catalytic mutant (TENT5C dCAT) reverted the expression of the endogenous transcripts to levels observed in the absence of TENT5C (Fig. 6B). These results showed that TENT5C corepressor activity of GR is not mediated by the LXXLL motif (3).

Given the interaction of TENT5C and ERα, we speculated that the LXXLL (3) motif could be required for the activity of TENT5C as a corepressor. To test whether the TENT5C LXXLL (3) motif mutation affects ERα function, we performed a reporter assay where we cotransfected HEK293T cells with ERα and TENT5C WT or TENT5C L3m expression plasmids in the presence or absence of E2. While TENT5C significantly reduced the ERE‐mediated transcriptional activation after E2 treatment, ERα activity was partially recovered when cotransfected with the TENT5C L3m mutant (Fig. 6C). To understand whether the dependency on the LXXLL (3) motif was cell‐type specific, we repeated the experiment in HepG2 cells stably expressing ERα. In this case, we observed no derepression of the reporter activity when the TENT5C L3m variant was cotransfected (Fig. 6D). To evaluate the repression activity of TENT5C on endogenous ERα target genes, we performed qPCR on progesterone receptor (*PGR*) and complement C3 (*C3*) transcripts. ERα transfection induced *PGR* and *C3* expression in the presence of E2, but when ERα was cotransfected with TENT5C, the expression levels of both genes decreased in the E2 treatment, showing that TENT5C is a corepressor of ERα in this context (Fig. 6E,F). Cotransfection with the TENT5C L3m expression plasmid resulted in a robust derepression of *C3* expression and to a lesser extent of *PGR* (Fig. 6E,F). The catalytically dead mutant did not affect the ERα corepressor activity of TENT5C (Fig. 6C–F). These results indicate that the TENT5C LXXLL (3) motif contributes to the formation of the TENT5C/ERα/E2 complex and significantly modulates the TENT5C‐mediated repression of ERα activity in a cell‐type specific manner.

## Discussion

TENT5C has been associated with tumor progression and the cellular response to steroids [7, 9, 10]. Here, we show that TENT5C is a corepressor of GR and ERα through distinct mechanisms. While TENT5C interacts with ERα via its LXXLL (3) motif, the motif is not sufficient to stabilize TENT5C interaction with GR according to our MD simulations. Instead, we found some evidence that TENT5C catalytic activity might contribute to the repression of GR (Fig. 6B). TENT5C depletion has been implicated in the altered susceptibility to DEX treatment. An initial study showed that TENT5C‐depleted MM cell lines have increased cell survival upon treatment with DEX [9]. However, a subsequent CRISPR screening showed that deletion of TENT5C increases the susceptibility of the cells to DEX [10]. A third study showed no increase in apoptosis upon DEX treatment in MM cell lines [5]. We speculate that these differences could be in part explained by the presence of different coregulators, other than TENT5C, in the different systems used.

Changes in the estrogen pathway signaling in the absence of TENT5C have been previously reported as well [9]. Importantly, ERα and GR signaling can influence each other [31, 32]. In breast cancer and endometriosis models, chromatin immunoprecipitation assays of the ERα and GR receptors have shown GR binding to ERα target genes when cells were simultaneously exposed to DEX and E2 [31, 32]. In endometriosis, DEX acts as an anti‐inflammatory, pro‐apoptotic factor, while estrogen facilitates proliferation. However, simultaneous treatment with E2 and DEX leads to GR binding to ER targets, changing DEX activity from pro‐apoptotic to pro‐survival [31]. Thus, the regulation of TENT5C targets might be further shaped by the interaction between the GR and ERα pathways. Moreover, the role of TENT5C as a coregulator of other NRs such as ERβ remains to be established.

RNA 3′ end processing by terminal nucelodidyltransferases (TENTs) and other RNA binding proteins is required for proper cell differentiation, early embryogenesis, fertility, and innate immunity [33, 34, 35, 36, 37, 38, 39]. Thus, dysregulation of TENT activity has been linked to diverse phenotypic outcomes [2, 4, 5, 6, 40, 41, 42, 43, 44, 45]. TENT5C‐deficient animals are viable, but males are sterile due to defective spermiation, the process by which spermatids are released from the seminiferous epithelium [2]. A whole testis RNA profile, however, shows almost no changes in gene expression between TENT5C null animals and controls. C3, a prominent ERα target, is among the eight transcripts dysregulated [2]. In this study, we show that TENT5C robustly blocks C3 transcriptional activation by ERα from a fourfold increase back to basal levels, and the repression is completely recovered by the L3m loss of function mutation. Changes in C3 expression in TENT5C null animals take place in an estrogen‐independent manner. Thus, TENT5C regulation of C3 expression could also be mediated in the absence of ERα.

TENT5C has been associated with multiple cancer types in addition to MM, with a correlation between low TENT5C expression and poor prognosis of most tumor types [46, 47, 48, 49]. Despite these results, the specific mechanisms of TENT5C‐mediated cancer suppression are only recently beginning to be elucidated. TENT5C mutations in human myelomas are distributed throughout most of the protein, including mutations to the LXXLL (3) motif and a mutation to a critical aspartic acid required for the catalytic activity of the protein found in different patients [50, 51].

TENT5C has been proposed to repress cancer progression through mechanisms independent of its catalytic activity. TENT5C directly interacts with polo‐like kinase 4 (PLK4), a key regulator of centriole duplication [29, 47]. Depletion of TENT5C leads to enhanced cell invasion in a PLK4‐dependent manner [47]. Disruption of the PLK4 and TENT5C interaction by mutating key residues in TENT5 also increases cell proliferation [29]. However, inactivation of the catalytic activity of the cells in the same system leads to a similar increase in cell proliferation, suggesting that both the structural and enzymatic properties of TENT5C are required in this context [29].

TENT5C function as a tumor suppressor in MM has been associated with its capacity to reduce the unfolded protein response (UPR) [4, 9]. TENT5C preferentially polyadenylates transcripts targeted to the endoplasmic reticulum [4]. The longer the poly(A) tail of the transcripts, the more they are translated, and the higher the protein load the endoplasmic reticulum must sustain. By mutating TENT5C, the endoplasmic reticulum load decreases in cancerous cells, which become less susceptible to UPR‐triggered apoptosis. Our data suggest that the poly(A) polymerase activity of TENT5C could also regulate the response to glucocorticoids to modulate the response to cancer. Any crosstalk between the UPR and NR pathways mediated by TENT5C remains to be established.

## Materials and methods

### Reagents

Dexamethasone (DEX, CAS ID: 50‐02‐2) and 17β‐estradiol (E2, CAS ID: 50‐28‐2) were purchased from Millipore Sigma (Burlington, MA, USA). All 10 mm stock solutions were prepared using dimethyl sulfoxide (DMSO) and kept at −20 °C.

### Cell lines

HEK293T (RRID:CVCL_0063), HepG2 (RRID:CVCL_0027), and HeLa (RRID:CVCL_0030) cell lines were purchased from the American Type Culture Collection (Manassas, VA, USA). Mycoplasma‐free cells were used for all experiments. Dulbecco's modified Eagle's medium (DMEM) (Invitrogen, Carlsbad, CA, USA) with 10% fetal bovine serum (FBS; Gemini Bio‐Products) supplement and 4 mm l‐glutamine (Invitrogen) was used to maintain HEK293T and HeLa cells. Phenol red‐free minimum essential medium (MEM) (Invitrogen) with 10% fetal bovine serum (FBS; Gemini Bio‐Products, West Sacramento, CA, USA) supplement and 4 mm l‐glutamine (Invitrogen) was used to maintain HepG2 cells. For hormone treatments, 10% charcoal/dextran‐stripped FBS (sFBS; Gemini Bio‐Products) was used.

### Plasmids

The pcDNA expression vector was purchased from Invitrogen (#V79020). The pCDH‐CMV expression vector, a gift from Kazuhiro Oka, and pcDNA‐TRBP expression plasmid, a gift from Dong‐Yan Jin, were obtained from Addgene (Cambridge, MA, USA) (#72265 and #15666) [52]. The internal control plasmid for transfection efficiency, pRL‐TK Renilla luciferase reporter vector (pRL‐TK‐luc) was purchased from Promega (Madison, WI, USA) (#E224A). The 2xGRE‐TATA fused to a pGL3 luciferase reporter plasmid (2xGRE‐luc) was obtained from Dr Robert Oakley and the mouse GR expression plasmids from Dr Tatsuya Sueyoshi [53, 54]. The synthetic vitellogenin 3xERE‐TATA fused to a pGL3 luciferase reporter plasmid (3xERE‐luc) and pcDNA/mouse ERα expression plasmids used in this study have been previously described [55]. The GFP‐ and HA‐tagged mTent5a (TENT5A) and mTent5c (TENT5C) expression plasmids were cloned into the pCDH‐CMV vector by GenScript Biotech (Piscataway, NJ, USA). The GFP‐ and HA‐tagged mTENT5C LXXLL (3) motif mutation, LXXAA (TENT5C L3m), was cloned into the BsiWI/SacII sites of the pCDH‐CMV vector (LXXLL: CTC atc tct CTC CTG and LXXAA: CTC atc tct GCC GCT). The GFP‐ and HA‐tagged mTENT5C catalytic mutant, dCAT (TENT5C dCAT), was cloned into the BsiWI/SacII sites of the pCDH‐CMV vector (DLD, GATttgGAT to ALA, GCTctgGCT).

### Luciferase reporter assay

Cells (140 000 per well) were seeded in 24‐well plates with 10% FBS medium and grown overnight. The next day, a total of 0.7 μg of expression vectors, which included 0.1 μg of pRL‐TK‐luc, 0.2 μg of 3xERE‐Luc or 2xGRE‐Luc, and 0.4 μg of expression plasmid, were transiently transfected using Effectene transfection reagent (QIAGEN, Germantown, MD, USA) according to the manufacturer's protocol. Six hours after the transfection, the medium was changed to 10% FBS in HEK293T and HeLa cultures or 10% sFBS in HepG2 culture. After a 20‐h incubation, all cells were treated with vehicle (70% EtOH) or receptor ligands (100 nm DEX or 10 nm E2) using 10% sFBS medium for 18 additional hours. The luciferase assays were performed using the Dual‐Luciferase Reporter Activity System (Promega). Transfection efficiency was normalized using the Renilla luciferase activity encoded in the pRL‐TK‐luc plasmid. Fold increases were calculated relative to each vehicle.

### Protein alignments

The following protein sequences were used for protein alignments: mouse TENT5A, NP_001153850; mouse TENT5B, NP_780516; mouse TENT5C, NP_001136424; mouse TENT5D, NP_001156576.1; human TENT5C, NP_060179. The protein alignment was done using muscle version 3.8.1551 [56].

### MD simulations

#### TENT5C simulations

The initial structure of human TENT5C was obtained from the X‐ray crystal structure (pdb ID 6W36); the missing residues 121–123 and 161–162 were added using modeller‐10.2 [29, 57]. The final model contained TENT5C residues 14 to 343 (Fig. S1). The method used to generate the simulations has been previously described [58]. For the preparation of starting configurations of molecular dynamics trajectories, missing atoms and protons were introduced using the leap module of amber.20 [59]; counter ions were added, and the systems were solvated in a box of TIP3P water with the box boundary extending to 20 Å from the nearest peptide atom, resulting in 107 405 atoms within the simulation box. Histidine residues 207 and 324 were δ‐protonated, while the rest of the histidine residues were ε‐protonated. There were 71 Na^+^ and 62 Cl^−^ ions providing charge neutralization and a 100 mm effective salt concentration close to physiological conditions. Prior to equilibration, the solvated system was sequentially subjected to (a) 500 ps belly dynamics with fixed peptide, (b) minimization (5000 steps), (c) low temperature (200 °K) constant pressure dynamics at fixed protein to assure a reasonable starting density (~ 1 ns), (d) minimization, (e) step‐wise heating MD at constant volume (from 0 to 300 °K in 3 ns), and (f) constant volume simulation for 20 ns with a constraint force constant of 10 kcal·mol^−1^ applied only on backbone heavy atoms. After releasing all constraining forces within the 30 ns equilibration period, sampling was increased by performing three independent, constant‐temperature constant volume MD simulations for 1 μs each. All trajectories were calculated using the PMEMD module of amber.20 with a 1 fs time step. Long‐range coulombic interactions were handled using the PME method with a cut‐off of 10 Å for direct interactions. The amino acid parameters were selected from the FF14SB force field of amber.20.

#### Simulations of TENT5C with ERα or GR complexes

The initial structure of the human ERα bound to an LXXLL motif‐containing peptide was obtained from pdb ID 1GWR; missing residues 332–334 and 462–464 were added using modeller‐10.2 [30, 57]. ERα residues 306 to 549 were present in the final model (Fig. S2). The initial X‐ray crystal structure with ERα and the bound peptide was used to guide the alignment of the TENT5C LXXLL motifs. Only the alignment of the LXXLL (3) motif produced a conformation of the TENT5C and ERα complex with no steric clashes, and this structure was subjected to MD simulations. There were 48 210 water molecules solvating the system with the box boundary extending 20 Å from the nearest protein atom. In addition, 103 Na^+^ ions and 88 Cl^−^ ions provided the neutralization and the 100 nm effective salt concentration. For GR, the initial structure was taken from pdb ID 1m2z (Fig. S2) [60]. DEX, the bound ligand in the structure, was also included in the model. Like for ERα, the LXXLL motif (3) was aligned to the bound peptide in the X‐ray structure to create the TENT5C/GR complex.

The preparation and dynamics steps were similar to the ones given above, and here again, three independent constant‐temperature constant volume MD simulations were performed for 1 μs each. Configurations selected at each nanosecond of the production runs were used in all the analyses using the Cpptraj module of amber.20. Using the MM/PBSA protocol of amber.20 [59], interaction‐free energies of TENT5C residues with ERα and vice versa were calculated for the 1000 configurations extracted at each nanosecond interval from each trajectory (totaling 3000 configurations). The ionic strength for MM/PBSA calculations was selected to be 0.1 m. All structural figures and videos from the trajectories were made using the software chimera‐1.16 [61]. An additional set of MD runs were performed for TENT5C L3m with ERα. The original wild‐type simulation system was modified only at the LL residues mutated to AA while the rest of the system remained intact. The MD simulations of this system were carried out in triplicates, and the analysis was identical to the one described above for the wild‐type system.

### Co‐immunoprecipitation (co‐IP)

HEK293T cells (2 000 000 per dish) were plated in 100 mm dishes overnight. Cells were transfected with 4 μg of expression vector using Effectene transfection reagent (QIAGEN) for 36 h. Cell lysates were prepared in 1 mL of Pierce IP lysis buffer (ThermoFisher, Carlsbad, CA, USA), and protease inhibitor cocktail was added. The protein lysates were immunoprecipitated by rocking with 50 μL of GFP nanobody (directly chemically linked to the Sepharose) for 2 h at 4 °C [62]. The GFP‐tagged TENT5 protein complexes were bound to Sepharose. The bound complexes were washed 5 times with HIP buffer (50 mm Tris‐Cl, 150 mm NaCl, and 1% Triton X‐100) and then boiled in SDS sample buffer. The immunoprecipitated proteins were then resolved on a 10% polyacrylamide Tris‐glycine gel. The proteins were transferred onto nitrocellulose membranes, and the membranes were subsequently blocked in PBS with 5% nonfat milk for an hour. The presence of ERα in the precipitants was detected with an anti‐ERα antibody (MC‐20, Cat# sc‐542, Santa Cruz, Santa Cruz, CA, USA). The presence of HA‐tagged TENT5A, TENT5C, or ERα in 2% of input samples was detected using anti‐HA Tag (C29F4, Cat# 3724S, Cell Signaling, Danvers, MA, USA) or anti‐ERα antibodies. The presence of c‐Myc‐tagged GR in the precipitants was detected by anti‐c‐Myc (9E‐10, Cat# sc‐40, Santa Cruz). The presence of HA‐tagged TENT5C or TRBP in 2% of input samples was detected by anti‐HA Tag (C29F4, Cat# 3724S, Cell Signaling) or anti‐TRBP (Cat# 15753‐1‐AP, Proteintech, Rosemont, IL, USA). The immunoreactive products were detected with the ECL Plus Detection System using the Amersham ImageQuant 800 system.

### qPCR assay

HeLa or HEK293T cells (200 000 per well) were seeded in 6‐well plates with 10% FBS phenol red‐medium and grown overnight. A total of 0.5 μg of expression vectors was transiently transfected using Effectene transfection reagent (QIAGEN) according to the manufacturer's protocol. A day after transfection, cells were changed to fresh starving medium for continued culture for 6 h. Then, they were treated with vehicle (70% EtOH) or receptor ligands (100 nm DEX or 10 nm E2) for 4 h in the case of the reporter assays and 18 h to assess the expression of the endogenous targets.

Total RNA was extracted from cells after transfection and ligand treatment using RNeasy Mini Kit (QIAGEN) according to the manufacturer's protocol. First‐stand cDNA synthesis was performed using Superscript Reverse transcriptase (Invitrogen). SYBR green assays (Applied Biosystems, Foster City, CA, USA) were used to measure mRNA levels of target genes. The qPCR conditions consisted of a denaturation step of 20 s at 95 °C followed by 40 cycles of 3 s at 95 °C and 30 s at 60 °C. Cycle threshold (Ct) values were obtained using ABI PRISM 7900 Sequence Detection System and analysis software (Applied Biosystems). The sequences of the primers used in real‐time PCR were as follows: for human *FKBP5* (NM_004117.4), forward primer 5′ AATGGTGAGGAAACGCCGATG 3′ and reverse primer 5′ TCGAGGGAATTTTAGGGAGACT‐3′; for human *PGR* (NM_000926.4), forward primer 5′ GGCTGTCATTATGGTGTCCTTACC 3′ and reverse primer 5′ CTGCGGATTTTATCAACGATGC 3′; for human *C3* (NM_000064), forward primer 5′ GGGGAGTCCCATGTACTCTATC 3′ and reverse primer 5′ GGAAGTCGTGGACAGTAACAG 3′. Each sample was quantified against its human *ACTB* (NM_001101) transcript content with forward primer 5′ GACAGGATGCAGAAGGAGATCAC 3′ and reverse primer 5′ GCTGATCCACATCTGCTGGAA 3′.

### Statistical analysis

The Dunn tests were used to analyze differences in Luciferase activity. Paired *t*‐tests were used to compare gene expression changes quantified by qPCR. The Hommel correction was used to correct for multiple comparisons as indicated in the figure legends.

## Conflict of interest

The authors declare no conflict of interest.

## Author contributions

YL and MM designed the study. YL performed most of the experiments and analyzed data with the help of RSH and MB. LP performed the MD simulation analysis. RMP contributed to the Co‐IP assay. YL, MM, RSH, and LP wrote the manuscript. MM supervised the study.

## Supporting information


**Fig. S1.** Simulation results of the solution structure of TENT5C alone.
**Fig. S2.** Human TENT5C, ERα, and GR structures.

**Video S1.** Video of a 1 μs long MD simulation of the TENT5C/ERα/E2 complex (sample 1).

**Video S2.** Video of a 1 μs long MD simulation of the TENT5C/ERα/E2 complex (sample 2).

**Video S3.** Video of a 1 μs long MD simulation of the TENT5C/ERα/E2 complex (sample 3).

## Data Availability

The data that support the findings of this study are available on request from the corresponding author.
