# Supplementary material for: TENT5C functions as a corepressor in the ligand‐bound glucocorticoid receptor and estrogen receptor α complexes
Source: FEBS J. 2025 May 27;292(19):5168–84. doi: 10.1111/febs.70137 (PMC12215964; doi:10.1111/febs.70137)
Supplement: Supplementary file 1 — Fig. S1. Simulation results of the solution structure of TENT5C alone. Fig. S2. Human TENT5C, ERα, and GR structures. [file FEBS-292-5168-s004.pdf]

*Supporting information*

# TENT5C functions as a corepressor in the ligand-bound glucocorticoid receptor and estrogen receptor α complexes

Yin Li[1], Lalith Perera[2], Rebecca S. He[1], Marine Baptissart[1], Robert M. Petrovich[2], and Marcos Morgan[1*]

[1]Reproductive and Developmental Biology Laboratory, National Institute of Environmental Health Sciences, Durham, North Carolina, USA

[2]Genomic Integrity and Structural Biology Laboratory, National Institute of Environmental Health Sciences, Durham, North Carolina, USA

*****Correspondence should be addressed to marcos.morgan@nih.gov

**Supporting Information**

**Supporting Figure 1. Simulation results of the solution structure of TENT5C alone.**

**Supporting Figure 2. Human TENT5C, ERα, and GR structures.**

**Supporting Video 1. Video of a 1 μs long MD simulation of the TENT5C/ERα/E2 complex (sample 1).**

**Supporting Video 2. Video of a 1 μs long MD simulation of the TENT5C/ERα/E2 complex (sample 2).**

**Supporting Video 3. Video of a 1 μs long MD simulation of the TENT5C/ERα/E2 complex (sample 3).**

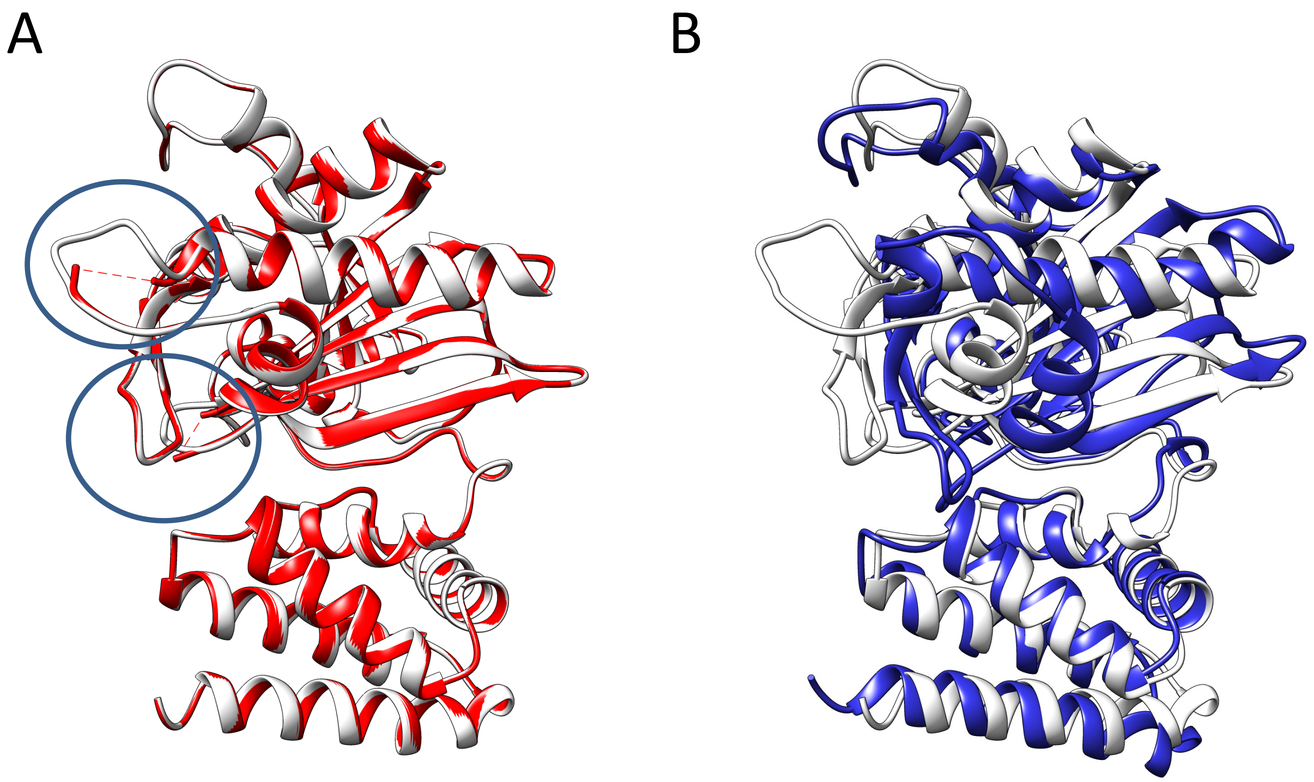


**Supporting Fig. 1. Simulation results of the solution structure of TENT5C alone.** (**A**) Crystal structure used in the simulations (in red) showing missing residues (dashed lines) that were modeled before the simulations (in white). The panel was generated using Chimera-1.16. (**B**) A representative conformation of the equilibrated solution structure of TENT5C (in blue) is aligned to the initial structure (in white). The panel was generated using Chimera-1.16.

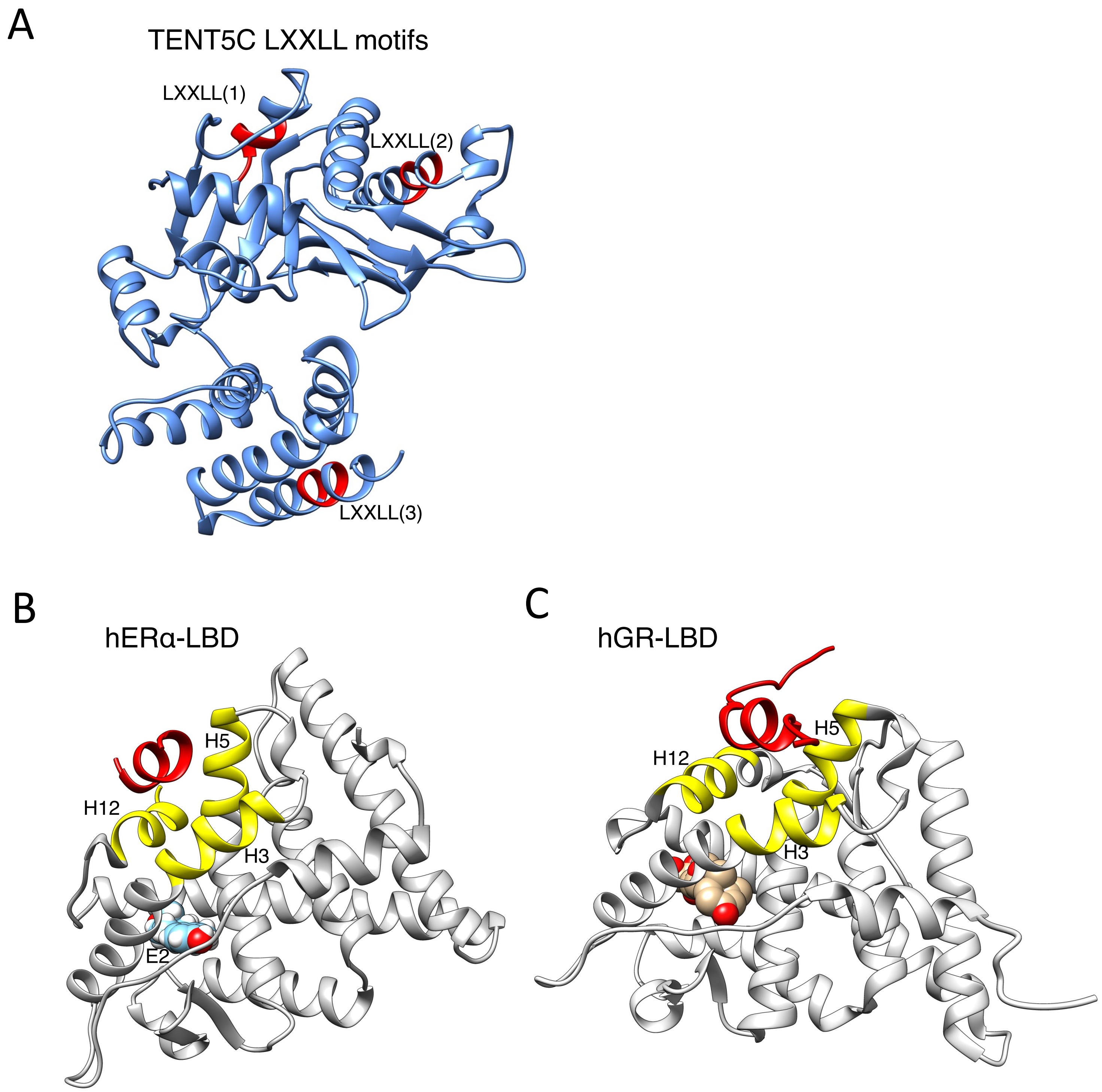


**Supporting Fig. 2. Human TENT5C, ERα, and GR structures.** (**A**) A representative solution structure of human TENT5C (residues 14-343) generated using Modeller showing the three LXXLL motifs in red. (**B**) ERα-ligand binding domain (LBD) structure generated with Modeller using PDB ID 1GWR. The bound coregulator peptide segment containing the LXXLL motif is shown in red. The three helical segments (H3, H5, and H12) making the coregulator LXXLL motif binding surface after the ligand binding to ERα are shown in yellow, and the ligand E2 is shown as solid spheres. (**C**) GR-LBD domain generated with Chimera-1.16 using PDB ID 1m2z. The peptide binding domain (in yellow) is shown together with the bound peptide (in red) and the ligand, DEX (in solid spheres).
